# Vibration-Based Wear Condition Estimation of Journal Bearings Using Convolutional Autoencoders

**DOI:** 10.3390/s23229212

**Published:** 2023-11-16

**Authors:** Cihan Ates, Tobias Höfchen, Mario Witt, Rainer Koch, Hans-Jörg Bauer

**Affiliations:** 1 Institute of Thermal Turbomachinery, Karlsruhe Institute of Technology (KIT), 76137 Karlsruhe, Germany; 2 Rheinmetall AG, Business Unit Bearings, KS Gleitlager GmbH, 68789 Sankt Leon-Rot, Germany; mario.witt@de.rheinmetall.com

**Keywords:** vibration sensors, journal bearings, state space modeling, state trajectory, machine learning, convolution, convolutional autoencoder, unsupervised learning

## Abstract

Predictive maintenance is considered a proactive approach that capitalizes on advanced sensing technologies and data analytics to anticipate potential equipment malfunctions, enabling cost savings and improved operational efficiency. For journal bearings, predictive maintenance assumes critical significance due to the inherent complexity and vital role of these components in mechanical systems. The primary objective of this study is to develop a data-driven methodology for indirectly determining the wear condition by leveraging experimentally collected vibration data. To accomplish this goal, a novel experimental procedure was devised to expedite wear formation on journal bearings. Seventeen bearings were tested and the collected sensor data were employed to evaluate the predictive capabilities of various sensors and mounting configurations. The effects of different downsampling methods and sampling rates on the sensor data were also explored within the framework of feature engineering. The downsampled sensor data were further processed using convolutional autoencoders (CAEs) to extract a latent state vector, which was found to exhibit a strong correlation with the wear state of the bearing. Remarkably, the CAE, trained on unlabeled measurements, demonstrated an impressive performance in wear estimation, achieving an average Pearson coefficient of 91% in four different experimental configurations. In essence, the proposed methodology facilitated an accurate estimation of the wear of the journal bearings, even when working with a limited amount of labeled data.

## 1. Introduction

Predictive maintenance has emerged as a revolutionary and proactive approach to equipment maintenance, surpassing traditional reactive or time-based strategies. Instead of merely addressing equipment failures after they occur, predictive maintenance leverages advanced measurement technologies and data analytics to anticipate potential malfunctions. By adopting this proactive methodology, industries can achieve significant cost savings and an improved operational efficiency [1]. In the context of mechanical systems, journal bearings play a vital role due to their inherent complexity and criticality. These bearings facilitate smooth and reliable rotational motion between two surfaces, effectively reducing friction and supporting heavy loads. However, continuous usage and operating conditions subject these bearings to wear, making them susceptible to failure if not properly monitored. For instance, in modern large wind turbines, journal bearings have replaced roller bearings to accommodate the constantly changing operating conditions of wind farms. This necessitates regular inspections and maintenance to ensure reliable turbine operation, accounting for a substantial 44–55% of the total operating costs within a mere 10 years [2]. Moreover, bearing damage contributes to up to 70% of cases involving longer downtimes, necessitating repairs of gearboxes, rotors, or generators [2]. A similar effect is also seen in engine failures [3]. To address these challenges and improve reliability, predictive maintenance becomes essential in enabling engineers to identify early signs of wear and estimate the remaining useful life of the bearing before it reaches a critical state. This capability allows for timely maintenance interventions, such as lubrication adjustments or replacements, effectively preventing catastrophic failures, costly repairs, and unplanned downtime.

The pursuit of in situ wear estimation has emerged as a promising technique for achieving the objectives of predictive maintenance. Conventional approaches to wear assessment often require the disassembly of machinery and meticulous inspections within controlled environments. In contrast, recent advancements in sensor technology have enabled a more proactive and non-intrusive approach to monitoring the condition of machinery during active operation. By strategically integrating various sensors into the assembly, real-time data related to key performance indicators can be collected. These sensors encompass a range of technologies, including vibration sensors, acoustic emission sensors, temperature sensors, and lubrication condition monitors, among others [4]. Of particular value are vibration sensors, which excel in detecting changes in the dynamic behavior of bearings [5], enabling the identification of abnormal vibration patterns associated with wear progression. On the other hand, acoustic emission sensors are adept at capturing high-frequency signals generated by emerging defects or wear-related stress, providing further insights into the health of the bearings [6].

In recent years, substantial attention has been dedicated to fault diagnosis in rotating machinery, with a significant focus on rolling bearings [7,8,9,10,11,12,13]. Studies pertaining to journal bearings, however, are fewer in number. A common approach is estimating whether the bearing operates in normal or abnormal conditions [14]. In some work, the abnormal conditions are classified into different types of abnormal condition such as Run-in, Steady1, Steady2, Pre-critical and critical [15], or the flow regime is classified from the measured data [16,17]. Other studies focus on areas such as the anomaly detection of force signals [18], the classification of operational states [15,17,19,20,21], load prediction [22], the estimation of model-based remaining useful life and wear prediction [23], and supervised wear volume estimation [24]. Data-driven regression models have been recently employed to assess the influence of temperature, bearing load, and rotational speed on the variation in friction torque and friction coefficient [25]. Bote-Garcia and Gühmann [26] used the integrated acoustic emission rooted-mean-squared value to estimate the wear state. They tested four journal bearings, with which they conducted six experiments (24 in total). During the experiments, the surface pressure and the rotation speed were held constant. After the data collection, different machine learning models were used for wear estimation: least square linear regression, random forests, multilayer perceptron (MLP), and gated recurrent unit-based recurrent neural networks (RNNs). In this particular study, the gated recurrent units outperformed the others with an $R^{2}$ value of 93%. For a more comprehensive review, interested readers are advised to refer to review papers covering bearing failure modes and their detection [27,28,29,30], data acquisition for fault detection [30,31,32,33], and machine learning applications for condition monitoring [34,35,36,37,38,39].

A literature analysis on predictive maintenance reveals that machine learning methods have proven effective in accurate bearing fault diagnosis, regime classifications, and wear volume estimations by leveraging collected sensor data. In particular, deep learning methods, such as convolutional neural networks and recurrent neural networks (RNNs), have demonstrated remarkable success in anomaly detection and regime classification, owing to their robust feature extraction capabilities [9,40]. However, it is important to acknowledge that these supervised learning methods rely heavily on large amounts of labeled data, which poses limitations in real-world applications. One of the primary challenges arises from the fact that bearings are designed to have a long lifespan, leading to a scarcity of cases with anomaly behaviors and high wear in the collected data. This creates an imbalance in the dataset, necessitating the collection of a significantly larger amount of training data during model development. Additionally, wear events occur at irregular intervals, prompting researchers to record vibration data at high sample rates to capture as much information as possible. However, this approach comes with the drawback of handling overwhelming volumes of data, which can be challenging. For wear estimation, another obstacle is that the initial gathered data typically contain only a single wear value—the one measured after dismounting the bearing. Unfortunately, due to the inability to precisely remount journal bearings in the exact same position, these data lack information density. As a consequence, an extensive amount of data must be collected for only one instance of the training set. Supervised learning-based methods also face the issue of domain shift [41]. In supervised learning, it is implicitly assumed that the training data (source domain) accurately represents the application environment (target domain), and the predictive accuracy of the model heavily relies on the representativeness of the source domain. However, in industrial settings, variations of operating conditions, sensor drift, environmental effects, and variations in other components of the rotating machinery can cause distribution discrepancies between sensory measurements collected for the same bearing conditions. More importantly, these measurements can be quite different from the examples in the training set that are at the same wear conditions. Consequently, when applying a model trained under controlled laboratory settings in the field, the diagnosis performance may be compromised. In light of these challenges, it is evident that novel approaches and techniques are necessary to address the limitations of supervised learning methods and ensure the successful implementation of predictive maintenance strategies in real-world scenarios.

In this context, our research introduces a novel approach that addresses these challenges and contributes to the field of predictive maintenance. We employ convolutional autoencoders as an unsupervised learning method to uncover meaningful temporal patterns within the collected sensor signals, eliminating the need for extensively labeled data. Furthermore, we have developed an innovative experimental procedure that accelerates wear formation on journal bearings, resulting in a more balanced dataset. By optimizing the feature engineering process and leveraging the extracted state space vectors, we construct wear state trajectories closely correlated with the actual measured wear, thus providing a robust solution for wear estimation. These contributions not only bridge the gap in supervised learning challenges, but also pave the way for effective predictive maintenance strategies in real-world scenarios.

The paper is organized into several sections. In the second chapter, we present the experimental setup, along with detailed descriptions of the measurement parameters. Subsequently, the Numerical Methods section elaborates on the feature engineering applied to the sensory data, outlines the feature extraction steps, and introduces the proposed methodology for wear state analysis. The results section is divided into two parts, complemented by two appendices. First, we present our findings on reducing the data complexity by the implementation of different metrics to assess signal similarity. Subsequently, we analyze encoded wear state trajectories as a means of estimating the wear of journal bearings.

## 2. Materials and Methods

### 2.1. Experimental Methods

Hydrodynamic journal bearings use viscous effects to generate a lubrication film via the no-slip condition at the fluid film body boundary at moderate speeds, i.e., operating below the critical Reynolds number. The bearing generates a pressure field due to this desired viscous effect and its rotation in an eccentric position caused by the applied load to the bearing. This pressure field acts as load-supporting mechanism. The integrated pressure field is the reaction force, which determines the bearing load-carrying capacity. At the same time, too much friction leads to wear and a further loss of functionality.

**Figure 1:**
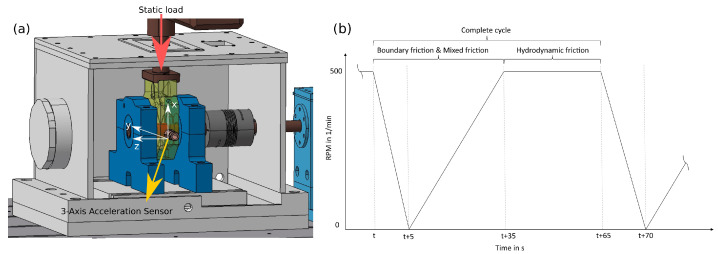
(**a**) CAD model of the experimental setup with the coordinate system. (**b**) Qualitative description of a cycle.

One of the main objectives in bearing development is the reduction in friction and wear to an absolute minimum. Hydrodynamic journal bearings especially have a very low friction coefficient at their operating point, as there are only viscous forces present. Consequently, hydrodynamic journal bearings have beneficial properties for machines that operate at fixed rotational speeds and loads. This advantage, however, is not useful in testing different designs and/or materials in an experimental campaign, as the regular operating mode will require a very large amount of time for wear to set in. For the current work, a friction-enhancing test rig was developed in order to accelerate the wear formation (Figure 1). The rig consists of a motor, a clutch, and a shaft, on which two support needle bearings are mounted. Between these bearings, the journal bearing that should be tested is mounted. A static 6 kN load is applied on it in a vertical direction to increase the wear. Additionally, the motor is driven with different rotational speeds in a cyclic scheme, as shown in Figure 1, which is motivated by the Stribeck curve [42]. Each cycle is 65 s long and consists of three phases. In the first five seconds, the rotational speed is decreased from 500 RPM to 0 RPM. Afterward, it is increased to 500 RPM over 30 s and held stationary for an additional 30 s. During the acceleration/deceleration phases, the journal bearing cannot create a supporting hydrodynamic film temporarily. As a result, mixed friction and boundary friction occur. In the stationary phase, a lubrication film supports the bearing, leading to only fluid friction, which has a low friction coefficient. Overall, conducting experiments at the mixed friction zone enables us to accelerate wear formation, hence reducing the overall experimental cost and the time needed to test multiple bearings.

**Figure 2:**
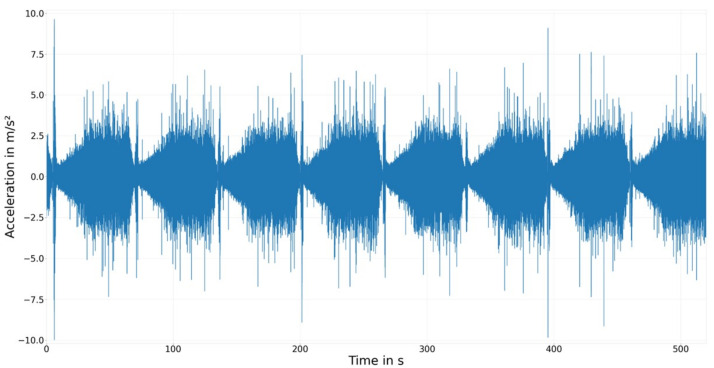
Cycle 1 to cycle 8 of Experiment 105 recorded with Sensor C.

For the reported work, we conducted 17 experiments (i.e., 17 bearings were tested) in total and each experiment comprised 900 cycles. Properties of the tested cylindrical bushes (referred as 30 × 33 × 20 mm) are given in Table 1. Before and after each experiment, the bearing was dismounted and the surface parameters were measured. A new bearing and a new shaft were used for each experiment. Wear detection was conducted indirectly via measuring vibrations. Five acceleration sensors were glued to the outer housing. Gluing was preferred to screwing in this case, as it eases the repeatability. Each sensor was sampled with 200 kHz. Figure 2 shows an example cycle, which clearly demonstrates the cyclic nature of the experimental procedure. Additionally, after each experiment, the wear was measured by measuring the diameter change of the bearing. It should be noted that during the experimental campaign, some components of the measurement setup (clutch, supporting bearings) were replaced, and/or sensors were remounted. Since such modifications alter the physical behavior of the whole setup (i.e., domain shift), the experiments were clustered as *Experimental setup*. The experiments of each group are conducted on the same testing rig without disassembling it. The only change that was performed between these experiments is the mounting and dismounting of the journal bearing and the shaft. The biggest challenge encountered throughout our experimental campaign pertained to the storage and post-processing of the collected sensor data. Each cycle within an experiment spanned 65 s, during which the sensors captured data at a rapid frequency of 200 kHz, resulting in a staggering 65 million floating-point data points per cycle. For an entire experiment, this aggregated to $5.85\times 10^{10}$ data points. This staggering volume of data motivated our exploration of alternative downsampling strategies. The primary aim was to minimize the memory footprint, as well as ensuring reasonable model complexity and efficient data processing in the subsequent stages of our machine learning implementation.

### 2.2. Data-Driven Methods

The primary objective of this study is to examine whether bearings with similar wear states exhibit comparable vibration pattern histories. We postulate that the acceleration measurements of each cycle can be represented as a state vector, and that the evolution of this vector concerned to a reference state reflects the wear history of the bearing. To achieve this, two critical tasks need to be addressed: (i) establishing an appropriate reference state, and (ii) quantifying the (dis)similarities between the reference state and individual cycles to trace the wear in the state space effectively.

**Figure 3:**
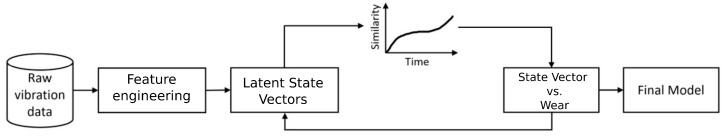
Proposed workflow to translate raw sensory measurements to state trajectories for wear estimation.

To assess the similarity, various metrics can be employed depending on the nature of the objects and the domain of study. Common similarity metrics include Euclidean distance, cosine similarity, Jaccard similarity, and others, each tailored to specific data types and applications. However, in high-dimensional spaces, these measures may lose their significance, necessitating feature engineering to overcome the challenges posed by the curse of dimensionality (the curse of dimensionality refers to the issues encountered when dealing with high-dimensional data spaces, where the number of features or dimensions grows significantly. As the dimensionality increases, the data space expands exponentially, resulting in data sparsity and data points being spread far apart from each other. This sparsity makes it difficult to identify meaningful patterns or relationships between data points) and improve the reliability of similarity assessments. Therefore, we adopt the approach illustrated in Figure 3. Initially, we reduce the dimensionality of the raw vibration data in three steps. Subsequently, the compressed state vectors are further transformed into latent state vectors using convolutional autoencoders. This transformation facilitates the quantification of the distance between each cycle and the reference state, resulting in a trajectory plot. In the final step, we compute Pearson correlations between the measured wear values after 900 cycles and the distances to the reference state at cycle 900. It is essential to note that wear measurements for intermediate cycles are unavailable, and the ground truth is known only at the experiment’s conclusion. As a result, we adopt a distance-based unsupervised approach to extract state trajectories, using the last measurement points to assess the distance-based approach’s capability to distinguish between different wear states. The subsequent sub-sections delve into the specific details of the downsampling policies, feature extraction using autoencoders, and the state trajectory calculations.

#### 2.2.1. Downsampling of Sensor Data and Cycle Similarity Analysis

The process of downsampling a digital signal aims to reduce its size using various methods. Let “n” represent the downsampling rate. The fundamental goal of downsampling is to compress consecutive slices of the original signal, each with a length of “n,” into smaller slices. In this study, we investigate three distinct approaches for downsampling, each resulting in scalar representations of the slices: (i) utilizing the median value of each slice from the absolute original data; (ii) employing the α-trimmed mean of each slice obtained from the absolute original data; and (iii) selecting every *n*th value of the absolute original signal.

After applying the downsampling techniques to the original signal, it becomes imperative to establish a metric that facilitates the comparison of the similarity between both the original and downsampled signals. This assessment is vital for evaluating the performance of each downsampling approach. However, given that the original signal and the downsampled signal differ in length, conventional similarity metrics like the Euclidean or Manhattan distances are not suitable. To address this discrepancy, we calculate the cumulative distribution function (CDF) for both signals. Subsequently, we measure the similarity between the resulting curves using the $L_{p}$ metric:

(1) $$L_{p}\left(X,Y\right):={\left(\int_{-\infty }^{\infty }{\left|F_{X}\left(x\right)-F_{Y}\left(x\right)\right|}^{p}dx\right)}^{\frac{1}{p}},\;p\geq 1,\;X,Y\in R^{1}$$

When p is equal to 1, the equation represents the $L_{1}$-metric, which was originally introduced by Kantorovich and Rubinstein in 1957. In 1969, Wasserstein utilized this metric in the context of Markov processes, leading to its prominence as the Wasserstein metric [43]. Essentially, the Wasserstein metric quantifies the minimal effort needed to restructure the probability distribution of one entity to match the other distribution. This distance calculation finds its inspiration from solving the optimal transport problem. The explicit equation for this $L_{1}$-Wasserstein metric is given by:

(2) $$L_{1}\left(X,Y\right):=\int_{-\infty }^{\infty }\left|F_{X}\left(x\right)-F_{Y}\left(x\right)\right|dx,\;X,Y\in R^{1}$$

As the value of “p” increases, the influence of larger differences between the cumulative distribution functions (CDFs), denoted as $|F_{X}\left(x\right)-F_{Y}\left(x\right)|$, becomes more pronounced, approaching the Kolmogorov distance for the case when p is equal to infinity [44]:

(3) $$L_{\infty }\left(X,Y\right):=\sup\left|F_{X}\left(x\right)-F_{Y}\left(x\right)\right|,\;X,Y\in R^{1}$$

In this study, both the Kolmogorov distance and the Wasserstein distance are utilized to measure the similarity between the CDFs. The assessment of the similarity allows us to quantify the impact of the downsampling methods and the downsampling rate.

#### 2.2.2. Convolutional Autoencoders for Latent State Representation

An autoencoder is a tandem of two neural networks, namely encoder and decoder. They work together to find a lower-dimensional projection of the original data, which contain the essential information. In the forward pass, the encoder compresses the input data (X) into the latent space (X${}^{\prime }$), which has a lower dimension than the input space. After data compression, the decoder reconstructs the data to the same dimensions of the input (X${}^{\prime \prime }$) from the latent space representation (X${}^{\prime \prime }$) [45]. Training an autoencoder follows the same steps as in feed forward networks. The only difference is the loss calculation. Since an autoencoder is an unsupervised learning algorithm with the aim to reconstruct the original data from a lower space representation, the similarity of the reconstructed data (X${}^{\prime \prime }$) and the original data (X) is used as a loss. This choice is reasonable because if the decoder is capable of creating a good reconstruction from the latent space, the compression of the encoder has to be useful; and therefore, the information density of the latent space is higher than the original representation.

**Figure 4:**
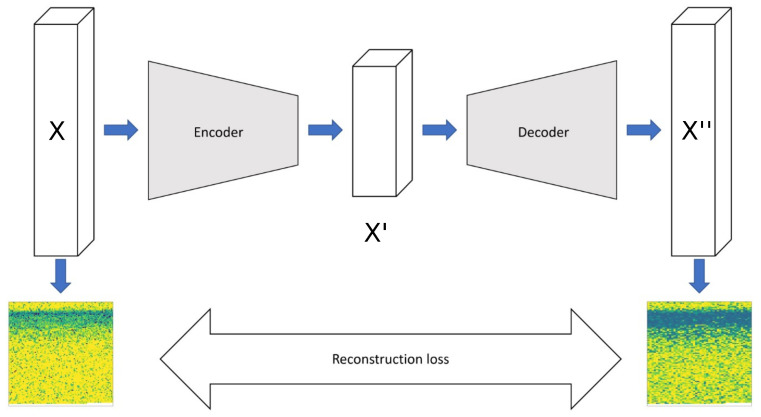
Illustration of the CAE model architecture with sensory measurements as single-channel measurements.

In this study, we employ a convolutional autoencoder (CAE) as a pattern recognition algorithm to analyze the collected sensory data (Figure 4). Our hypothesis is that local relationships between data points in the time series contain valuable information about the wear due to the stochastic and short-lived nature of wear-causing events, such as debris collisions. To capture these short-term time dependencies efficiently, we utilize convolution filters in time, which significantly reduce the number of model parameters.

**Figure 5:**
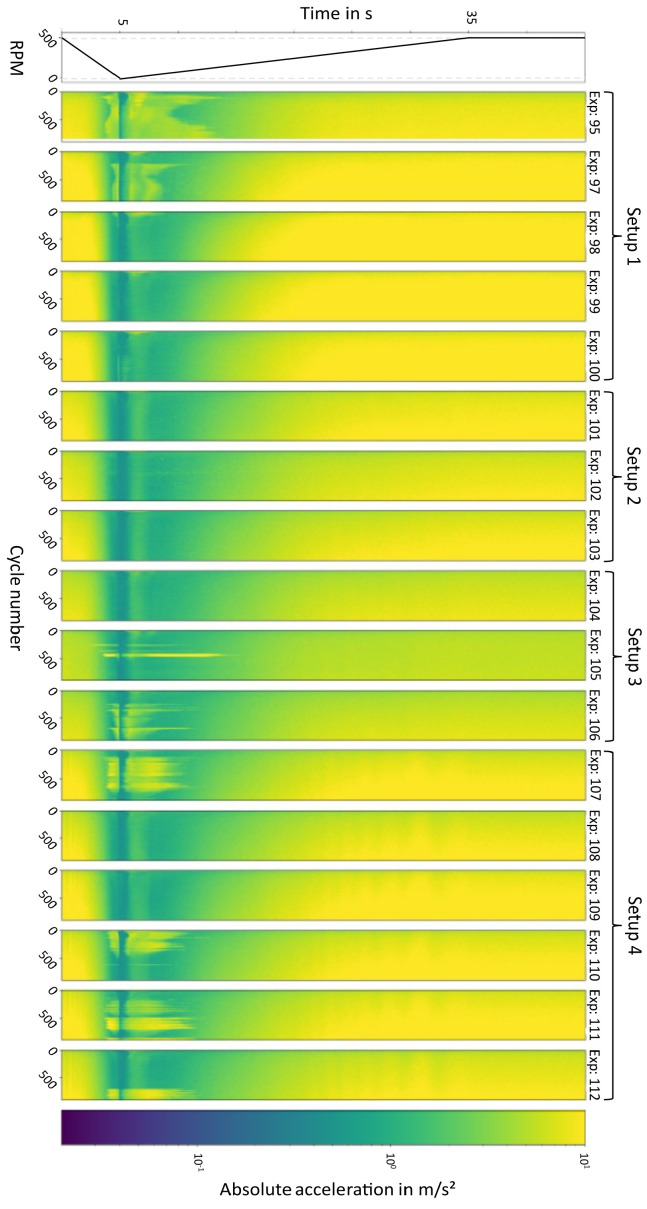
Acceleration measurements of sensor C for the 17 experiments. Each row of sub-figures depicts the absolute acceleration per cycle. Corresponding RPM is also given at the top. Setups 1–4 refer to the measurement clusters. See Experimental Methods for more details.

To use the CAE architecture, we first reshape the 1D time series data from the experiments into 2D matrices, where each row corresponds to one cycle (Figure 5). To determine the optimal model architecture, a preliminary study was conducted using a grid search. Details of the tested hyperparameters are given in Table 2. It should be noted that the number of layers needed for the CAE is computed for the set compression ratio, given the downsampling options (stride and pooling). The raison d’être for the further compression (i.e., dimensionality reduction) is to enforce representation learning within the convolutional network. Representation learning is crucial for state space trajectory analysis, as it enables the extraction of meaningful and compact features from the high-dimensional sensory data. These learned representations capture underlying patterns and temporal relationships within the data, allowing for a more effective interpretation and estimation of state trajectories, which is a fundamental aspect of our approach.

We aimed to treat cycles independently, so we employed 1D kernels to capture local, short time scale events with an increasing time window, ranging from (2,1) to (7,1). Additionally, we examined a range of kernel numbers from 16 to 512. Our main goal was to achieve a high compression ratio, and we tested three alternative methods for downsampling. In Method 1 (M1), we used strides greater than 1 in combination with *MaxPooling*. In M2, we solely relied on *MaxPooling* to reduce the array sizes. In M3, we tried a less conventional approach by applying median pooling to the kernel dimension rather than on the filtered images. Throughout all hidden layers, we used *ReLU* activation with *He initialization*. All models were created and evaluated using TensorFlow 2.0, with custom modifications as needed (e.g., median kernel pooling). To assess the model performance, we measured the similarity between the reconstructed input vectors and the original input image using two full-reference image similarity metrics: (i) the percentage of pixels with an absolute difference smaller than 0.01, and (ii) the Manhattan distance (see Appendix A for more details). For training and testing the CAE, we utilized 15,300 instances. This number corresponds to the amount of cycle data collected across 17 experiments. An example of such an instance is depicted in Figure 4 (model input X). If we concatenate all 900 measurements from a single experiment, we obtain one of the “stripes” as illustrated in Figure 5, where all the data collected from the 17 experiments (a total of 15,300 cycles) along with their corresponding labels can be found.

**Figure 6:**
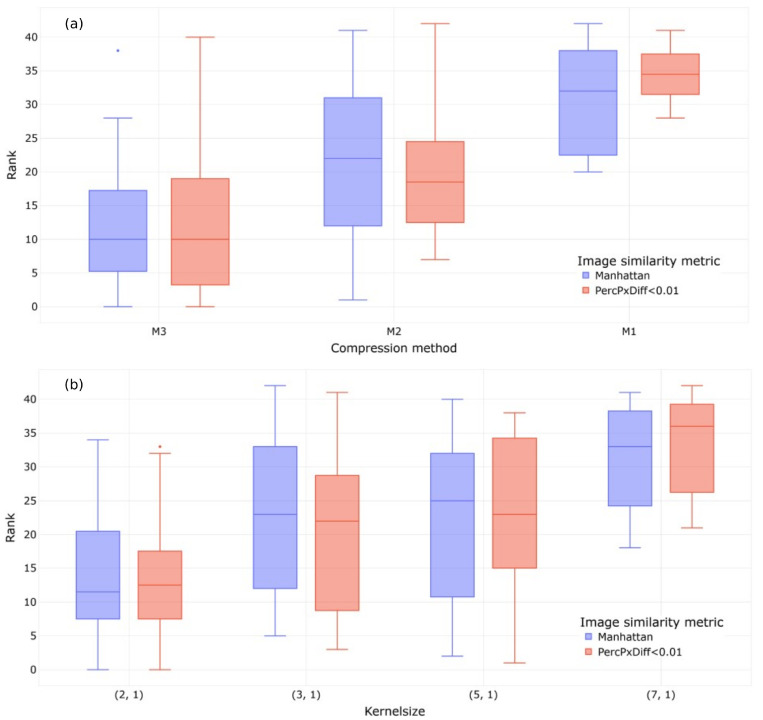
(**a**) Performance evaluation statistics of different compression methods. (**b**) Performance evaluation of different kernel sizes. Sorted by the ranking in reconstruction accuracy (lower rank = better).

The findings of the parameter study are presented in Figure 6, where the y axis denotes the accuracy ranking statistics of a hyper-parameterized model for a given compression method, or the kernel sizes. Herein, the ranking variations are caused by the other two hyperparameters of the model (number of kernel, compression method, kernel size). It is seen that using kernel-wise median pooling (M3) and deploying smaller kernel sizes result in a better performance for a compression ratio of 2. The model parameters for higher-compression-ratio architectures were then analyzed for only M3 and a kernel size of (2,1). Finally, the representation learning capabilities of the 20 best models were compared within the state trajectory analysis (two models with a compression ratio (CR) of 2; 8 models with a CR of 4; and 10 models with a CR of 8).

#### 2.2.3. State Trajectory Analysis

The state vector representing one cycle remains high dimensional, even after applying the feature engineering steps and projecting the sensory data to a latent space using the highest-compression-ratio CAE (from 9 million to 1125). To obtain an interpretable 2D map of state trajectories, we calculated the distance between the state vectors and a reference state.

In the initial analysis, we investigated the influence of the reference state selection by computing the $L_{1}$ norm. The reference state can either be the origin (i.e., a zero vector of the same size) or the *n*th cycle of an experiment (i.e., first measurement, 100th measurement, or a randomly chosen cycle). After finding the impact of the reference state selection, we explored alternative statistical measures of the $L_{1}$ norm, including the median, the α-trimmed mean, and the interquartile range, to construct the state trajectory map. In all cases, the reference is taken as the origin. Among these measures, the one that exhibited the strongest correlation with accumulated wear was the maximum of the distance metrics.

For the α-trimmed mean:

(4) $$\begin{array}{c} {\bar{d}}_{\alpha ,i}=\frac{\sum_{j=q+1}^{n-q}\left(x_{j}-0\right)}{n-2q}\\ d_{i}=\max\left(d_{i-1},{\bar{d}}_{\alpha ,i}\right) \end{array}$$

And for the $L_{1}$ norm:

(5) $$\begin{array}{c} L_{1_{i}}=\sum_{j=1}^{n}\left|x_{j}-0\right|\\ L_{1_{i}}=\max\left(L_{1_{i-1}},L_{1_{i}}\right) \end{array}$$

Both of these descriptions demonstrated a strong consistency with the measured maximum wear value, effectively capturing short-lasting effects for future preservation, and will be utilized in the following sections. Additional details regarding the preliminary analysis and the rationale behind this selection are provided in Appendix B.

## 3. Results

### 3.1. Feature Engineering for Wear State Representation

For each cycle of the measurements, the experimental data include: (i) five acceleration measurements at 200 kHz frequency; (ii) measurements conducted on the tested bearing before the experiment, including surface roughness, skewness, kurtosis, and conicity; (iii) measured shaft features; and (iv) the clearance. Before testing alternative state representation models to build a wear trajectory, we first checked whether the additional features contain sufficient information about the final wear, and whether it is possible to downsample the sensor measurements and the number of sensors with minimal information loss.

**Figure 7:**
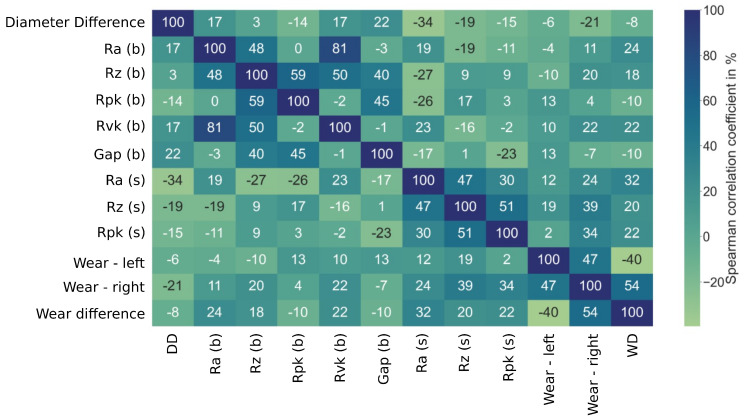
Spearman correlation coefficients between the measured initial parameters and the wear after 900 cycles. b and s refer to bearing and shaft parameters, respectively.

Figure 7 displays the Spearman correlation coefficients for each of the initial parameters measured and the resulting wear. One noticeable correlation is between the two bushing roughness values: Ra(b) and Rvk(b). When the correlations between the wear and the rest were examined, however, no strong correlations were found and these variables were dropped from the feature set.

**Figure 8:**
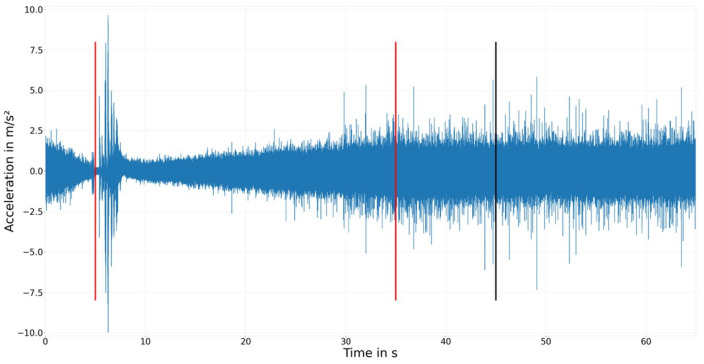
Example cycle of Experiment 105 recorded with Sensor C. Red lines mark the time with RPM; whether it is increased or decreased. The black line marks the cutoff point.

The next goal of feature engineering was to reduce the content of the sensor measurements with minimal information loss. Since each sensor samples at 200 kHz, a large amount of data are produced per cycle to be stored and processed. More importantly, the dimensionality of the cycle state becomes extremely large and is likely to suffer from a curse of dimensionality. Herein, dimensionality reduction was performed in three steps: (i) dropping uninformative sections of the cycle data via domain knowledge, (ii) dropping redundant sensor measurements, and (iii) downsampling the high-resolution temporal data via similarity analysis. The black line in Figure 8 marks the chosen cutoff point used to reduce the cycle length via domain knowledge. It was set after 45 s to ensure that the complete mixed friction zone is kept, as well as 10 s from the hydrodynamic friction zone. This choice was based on how journal bearings operate, that is, the information about the wear history contained in the cycle data should not change throughout the stationary phase, as almost no friction occurs there (see Experimental Methods for more details). Therefore, reducing the stationary phase length should not lead to a loss of information. The red lines mark the timestamps where the RPM is changed from decreasing to increasing and from increasing to stationary. A clear influence of the RPM on the vibration pattern is seen and the aforementioned three different stages (Figure 1) are clearly visible.

After the first reduction step, the dimensionality of the cycle state vector is reduced to 9 million per sensor, still leading to an exorbitant memory consumption per experiment. In the next step, the variances in the sensory measurements were compared to find out which sensor is the most informative. For that purpose, we established the following criteria:

- The acceleration values should exhibit a significant difference between earlier and later cycles of the same experiment.
- The measurement differences between two experiments should be substantial if the experiments exhibit different levels of wear.

**Figure 9:**
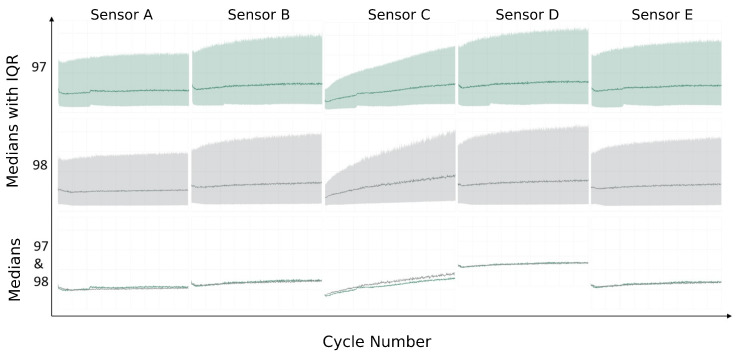
Median and interquartile range (IQR) of Experiment 97 and 98 for each sensor. Third row visualizes only the medians of Experiment 97 and 98.

Figure 9 illustrates how the the cycle medians and interquartile ranges evolve during Experiment 97 (19 µm wear after 900 cycles) and Experiment 98 (26 µm wear after 900 cycles) for each sensor. It is clearly seen that Sensor C best satisfies the aforementioned requirements: (i) the state of the journal bearing exhibits the desired upward trend of the utilized metric (see Section 2.2), (ii) the spread of the measured values per cycle grows in time, and most critically, (iii) a significant degree of separation emerges between experiments of different wear. Conversely, other sensors have a worse resolution in capturing the state evolution, as they do not effectively distinguish a 37% difference in wear.

**Figure 10:**
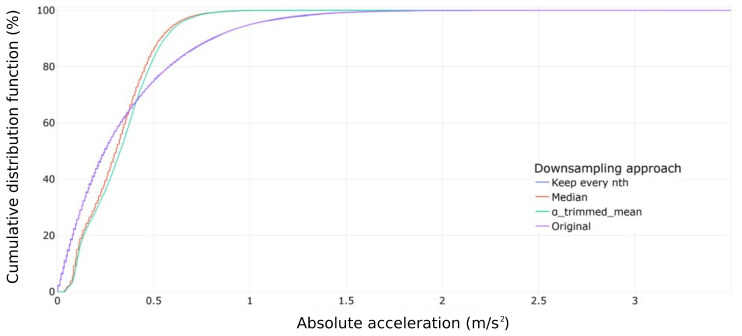
Cumulative density function of different downsampling approaches and original data for cycle 200 of Experiment 106. Downsampling factor: 1000. Sensor C.

**Figure 11:**
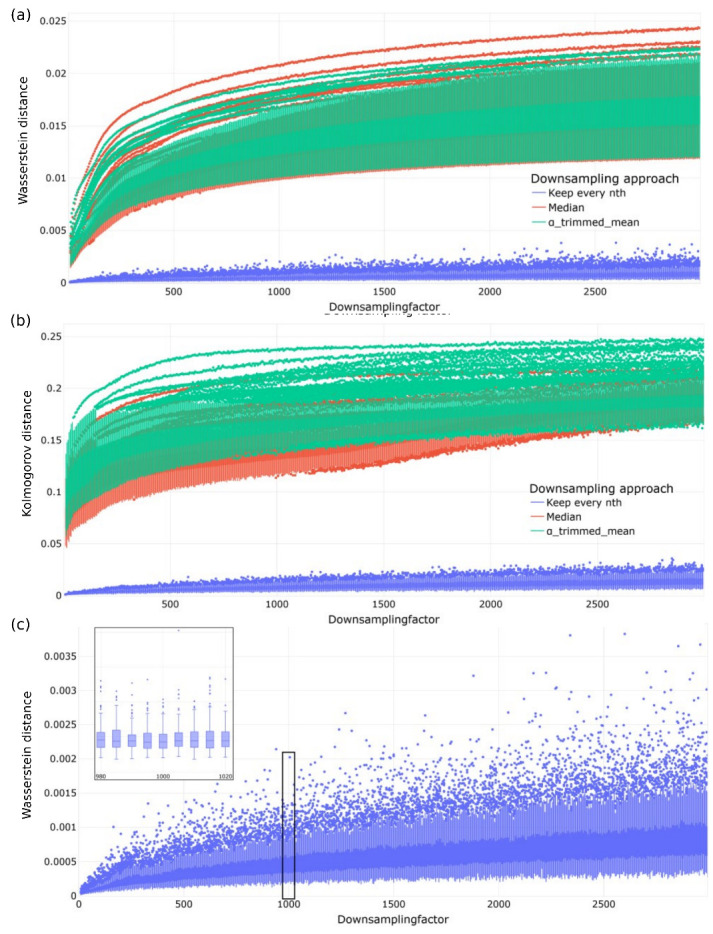
(**a**) Wasserstein and (**b**) Kolmogorov distance for all cycles of Experiments 105 and 106 for different downsampling factors. (**c**) A zoomed-in view on Box–Whisker plots for Experiment 105. Sub-figure on the upper-left corner shows the region highlighted with the black box.

In the third downsampling step, we investigated the optimal temporal resolution required for precise and reliable wear estimation. This investigation involved the implementation of three alternative downsampling policies (Section 2.2.1). Figure 10 illustrates the CDFs resulting from downsampling each approach by a factor of 1000. Additionally, Figure 11 presents the increasing dissimilarities between the original and downsampled signals as the downsampling rate rises. For each downsampling factor, distance values per experiment per distance metric were computed, and the results were analyzed using Box–Whisker plots (Figure 11). The comparisons revealed that the simplest downsampling approach, which retains every n-th value, outperforms the other methods. The distance metrics generally followed a linear trend, with very few outliers in cycle dissimilarity. Upon examining the distance curves for different experiments, a downsampling factor of 1000 was selected for its ability to simplify calculations. This choice is further supported by Figure 10, which indicates that the information loss from downsampling with a factor of 1000 is negligible.

### 3.2. Wear State Estimation via Convolutional Autoencoders

The feature engineering steps discussed in the previous section reduced the dimensionality of the state vector to 9000 per cycle. In the final step, we utilized CAEs to project the raw state vectors into a more compressed latent representation, using the trained encoder network. This latent space vector was then employed to construct the 2D wear state maps. Integrating CAEs in state trajectory mapping allowed us to benefit from their denoising and pattern extraction capabilities, leading to improved generalizability.

**Figure 12:**
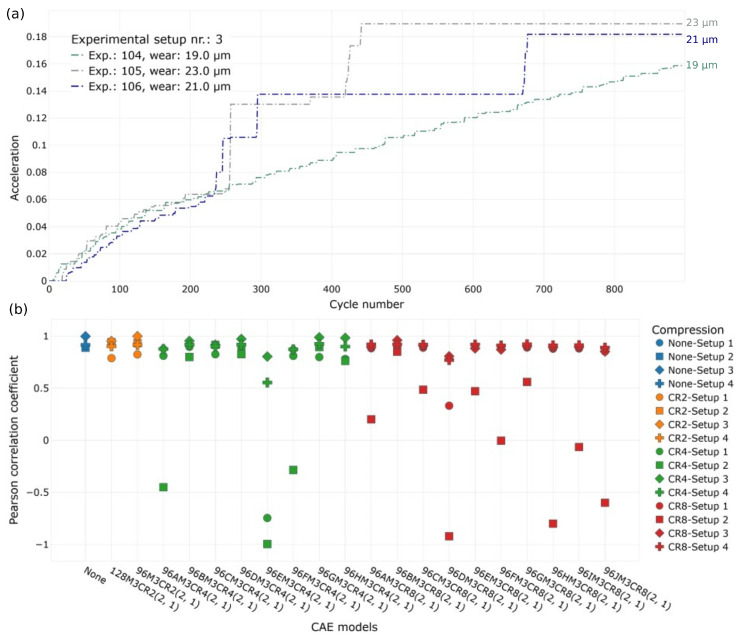
(**a**) Wear state trajectories calculated by using using 96BM3CR8(2, 1) CAE for Experimental Setup 3. The latent space vectors are converted to 2D map via α-trimmed mean (α = 10%) of the cycles. Pearson corr. coeff. is 96.1%. (**b**) Pearson correlation coefficient of different CAE models used for data encoding with different CRs. Model names are internal identifier.

In Figure 12a, we illustrate three state trajectories from Experimental Setup 3, using the CAE with a compression ratio (CR) of 8 as an example. Each curve in the graph represents the wear state for an experiment within Setup 3, with each data point indicating the state after cycle i. It is essential to note that “Experimental Setup” refers to a set of experiments conducted with a specific measurement setup, including components like the clutch, supporting bearings, and sensor mounts. Despite the bearings being the same within manufacturing tolerances, such modifications alter the physical behavior of the entire setup, leading to a domain shift. To account for these differences, we organized sub-groups of experiments to analyze the latent space trajectories within each group. However, the CAE model is trained on all 17 experiments, encompassing four experimental setups. This approach ensures that the feature extraction model can operate effectively regardless of domain shifts. As a result, the encoder network successfully distinguishes between the wear states of three different bearing tests, as demonstrated in Figure 12a.

In an attempt to create a quantitative comparison between different CAE model implementations (see Section 2.2.2 for model details), we calculated the Pearson correlation coefficient between the latent state vector, and the measured wear at the end of an experiment. This can be interpreted as a simple linear fit between the state vector encoded by the CAE and the label (measured wear after cycle 900). We followed such an approach, as the number of labeled cycle was very limited (17), compared to the total number of cycles (15,300). Figure 12b depicts the Pearson correlations for the top 20 CAE models. Herein, the Pearson correlation coefficient measures the strength and direction of a linear relationship between two continuous variables, i.e., the distance of the state vector to the origin and the wear. It ranges from −1 to +1, where +1 indicates a perfect positive correlation, −1 indicates a perfect negative correlation (as one variable increases, the other decreases), and 0 indicates that the variables are not linearly related. Most of the CAEs demonstrated a high predictive power regarding the wear state for all four setups (i.e., different hardware configurations). However, the figure also shows that as the CR increases, achieving a strong correlation becomes more challenging for the model. Interestingly, the correlation coefficient for Experimental Setup 3 remained consistently high for each model, demonstrating the effectiveness of the autoencoder in correctly encoding abrupt acceleration changes and removing continuous soft changes due to the limited dimensionality. This effect became more pronounced as the CR increased (see Figure 5 and Appendix B for more details about the sudden acceleration patterns observed in Experimental Setup 3). It is worth noting that some CAEs projected the state vectors in a mirrored symmetry, resulting in negative high correlations, particularly for Setup 2. The overall performance of the CAEs over four Experimental Setups is summarized in Table 3. For the highest compression ratio and the corresponding CAE model (96BM3CR8(2,1) in Figure 12), the average correlation coefficient is found to be 91%. It should also be noted that the inference time for the CAE was also relatively fast regardless of the CR (60 ms, averaged over 100 cycles) on a regular PC, highlighting the approach’s potential for product integration.

For our specific problem, our analysis has demonstrated that downsampling the raw sensor data by a factor of 1000 effectively preserves cycle data information with minimal loss. When dealing with different bearing tests, it is advisable to follow the same methodology (refer to Figure 9, Figure 10 and Figure 11) to determine an appropriate, case-specific downsampling factor. In the design of the CAE, using a kernel size of (2,1) combined with median pooling in the kernel dimension has proven to be more robust than employing strides and MaxPooling for downsampling. Furthermore, after training, the inference time for high compression ratios was negligible, all while maintaining a similar level of accuracy. This efficiency can be particularly valuable for lightweight implementations. Lastly, in the analysis of state space (dis)similarity, employing the $L_{1}$ norm has proven to be a robust and useful choice, especially when using zero vectors as a reference state to build a wear state map.

## 4. Conclusions

This study aimed to develop a novel metric for indirectly measuring the wear of a journal bearing using vibration sensors. A novel experimental procedure was established to accelerate the wear production by cyclically varying the motor speed. After examining various sensors and alignments, it was found that a single acceleration sensor is sufficient and the recorded data can be compressed by a factor of 1000 without losing much information. The study also revealed that the experimental setup has a significant influence on the recordings that cannot be compensated, so clusters of experiments were defined that correspond to unique hardware arrangements. Different methods for quantifying time series similarities were tested, and the maximum of the α-trimmed mean of a cycle was found to be the most suitable norm. The study further examined the use of a convolutional autoencoder (CAE) for compressing the sensory data to build a state trajectory map for wear estimation. Herein, median pooling in the kernel dimension increased the reconstruction capabilities of the CAE consistently. The CAE, trained on unlabeled measurements, demonstrated remarkable performance in wear estimation, achieving an average Pearson coefficient of 91% once the latent space representations were mapped via the maximum of the α-trimmed cycle mean values.

Overall, the proposed methodology allowed us to accurately estimate the wear value of the journal bearing based on limited labeled data. Our key findings highlight the successful application of convolutional autoencoders (CAEs) for feature extraction and wear estimation, across four distinct experimental setups. To overcome the challenge of imbalanced data, we introduced an innovative experimental procedure that accelerates wear formation, ensuring a more balanced dataset. Additionally, the study found that recording the journal bearing’s vibration signal while operating in the mixed-friction regime is crucial, as this regime contains important information about the current wear state. Measuring the vibration signal of only the fluid-friction regime lacks this information and is therefore insufficient for wear estimation.

## Figures and Tables

**Table 1:** Journal bearing properties.

Property	Value
Inner Bushing Diameter	30 mm
Wall Thickness	1.5 mm
Outer Bushing Diameter	33 mm
Bushing Width	20 mm

**Table 2:** CAE model hyperparameters.

Parameter	Tested Hyperparameters
Number of kernels	16, 32, 64, 128, 256, 512
Kernel size	(2,1), (3,1), (5,1), (7,1)
Stride	None, (2,1), (3,1)
Pooling	None, MaxPooling, MedianPooling, FilterwiseMedianPooling
Compression ratio	None, 2, 4, 8

**Table 3:** Pearson correlation coefficients of the best-performing CAE at each compression ratio (CR).

Compression	None	CR2	CR4	CR8
Minimum Pearson correlation	88.94%	82.46%	79.91%	85.08%
Mean Pearson correlation	92.36%	91.83%	90.22%	90.72%

**Figure A1:**
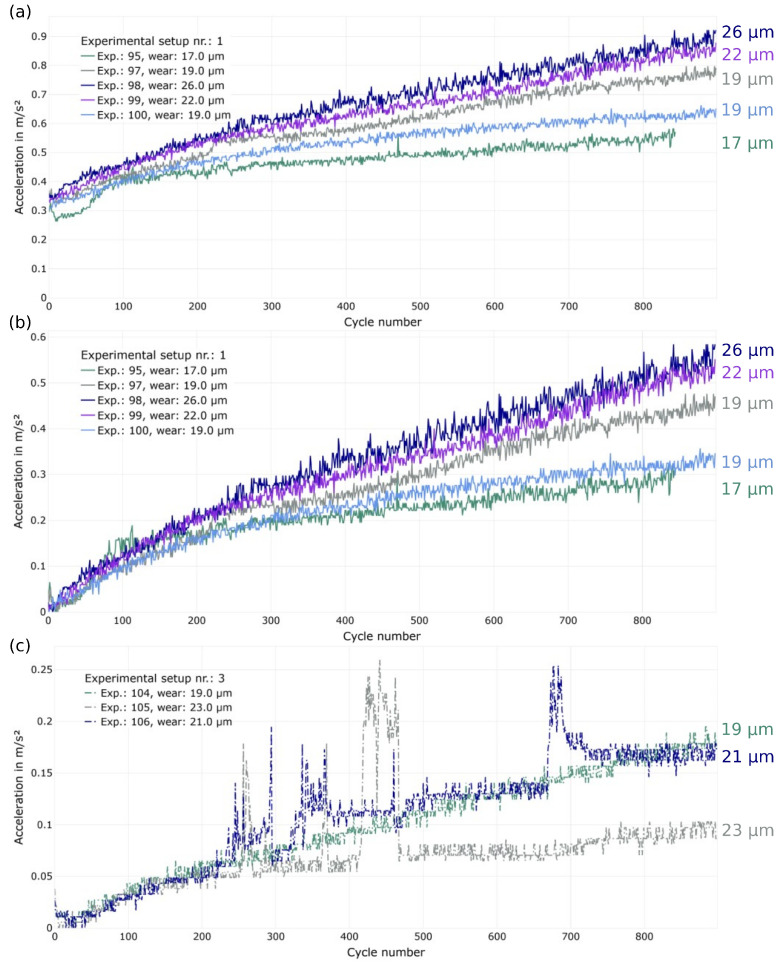
(**a**) Wear state trajectories calculated as the medians of the cycles for Experimental Setup 1. (**b**) Wear state trajectories calculated as the medians of the cycles with baseline correction for Experimental Setup 1. (**c**) Wear state trajectories calculated as the medians of the cycles with baseline correction for Experimental Setup 3.

**Figure A2:**
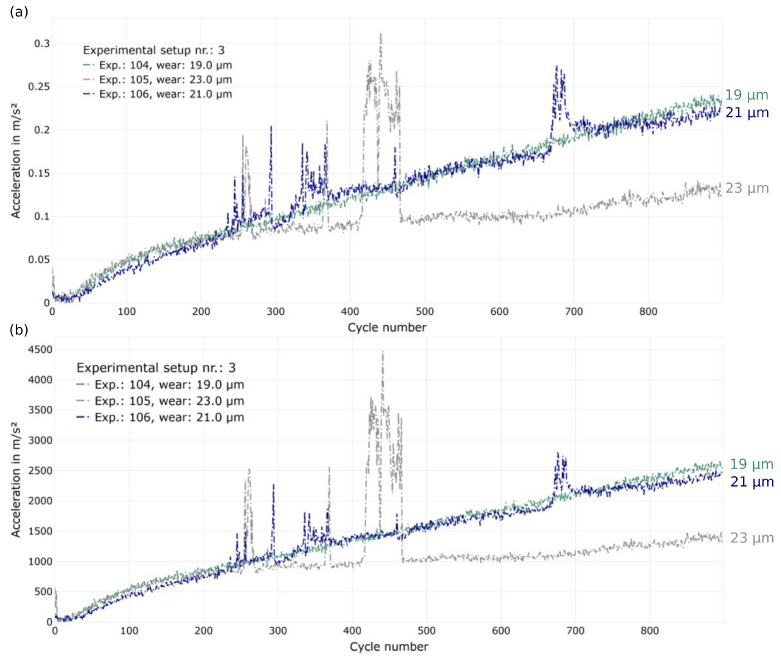
(**a**) Wear state trajectories calculated as α-trimmed mean (α = 10%) of the cycles for Experimental Setup 3. (**b**) Wear state trajectories calculated as $L_{1}$ norm of the cycles for Experimental Setup 3.

**Figure A3:**
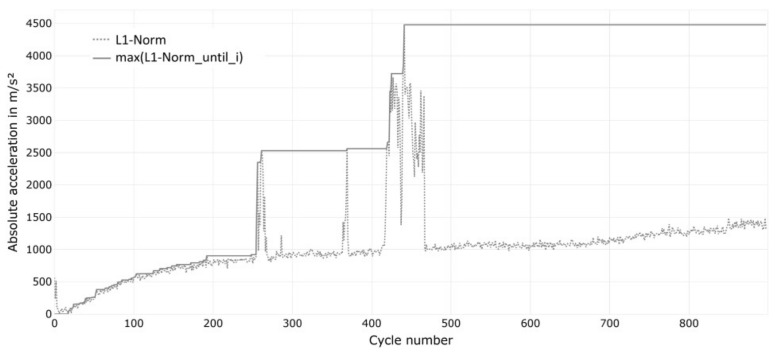
Wear state trajectories calculated as $L_{1}$ norm of the cycles for Experimental 105.

**Figure A4:**
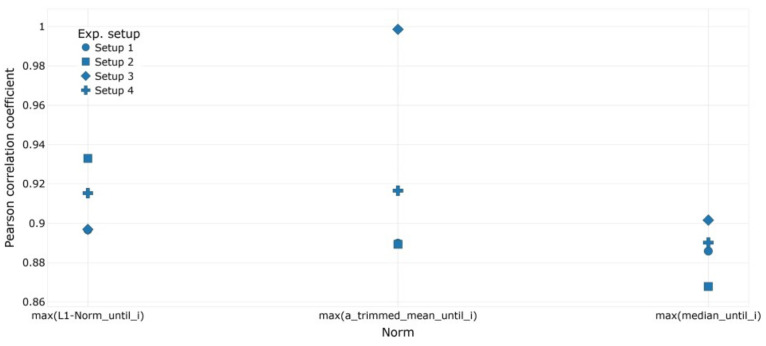
Pearson correlation coefficient of the adapted norm (cycle = 900) and the measured maximum wear value.

## Data Availability

The data are not publicly available due to privacy and confidentiality concerns.

## Appendix A. Image Similarity Metrics

Image similarity metrics can be categorized into subjective and objective metrics [46]. Subjective metrics aim to mimic the human visual system, which is considered the ultimate receiver of visual signals. However, while subjective metrics are considered reliable and accurate, they are not practical for optimization in real-world systems due to their time-consuming and expensive nature. Objective metrics, on the other hand, can automatically assess image quality, making them suitable as loss functions.

Objective image quality metrics (IQA) can be categorized into three types: full-reference IQA, reduced-reference IQA, and no-reference IQA. Full-reference IQA measures image similarity based on a complete reference image. Reduced-reference IQA measures overall image similarity using a reduced view of the image. No-reference IQA aims to predict image quality without an original reference image, often by measuring artefacts. Since full-reference IQA is more reliable than reduced-reference IQA, and no-reference IQA algorithms lack the ability to compare two images, we tested alternative full-reference IQA (FR-IQA) during the preliminary assessments.

FR-IQA is a method that predicts the similarity between two images by comparing the full images. After preprocessing the images, features are extracted from them, and their distance is calculated to determine the image similarity, which represents the reconstruction quality score. The feature extraction process can occur either in the spatial domain, in the transform domain, or in both. Commonly used spatial domain approaches include signal fidelities (MSE, MAE, RMSE, PSNR), structural similarity and variants (SSIM), distortion-based methods (UQI), and data-driven feature extraction and integration methods [47,48]. During the CAE model hyperparameter tuning, similarity metrics were first compared by using correlation matrices to decide which metrics are redundant. Most of the metrics yielded high correlations with one another. In accordance, we used (i) the percentage of pixels with an absolute difference smaller than 0.01 and (ii) the Manhattan distance to assess the reconstruction loss as they are easy to interpret.

## Appendix B. Distance Metric Selection for State Trajectory Analysis

To explore cycle similarity, our initial approach involved comparing statistical values such as the median, the α-trimmed mean, and the interquartile range for each cycle. This method has the advantage of being resistant to outliers, as these straightforward statistical measures are not heavily influenced by extreme data points. Figure A1 displays the medians of cyclic experiments conducted within the cluster *Experimental Setup 1*. The figure reveals a direct correlation between the median of the absolute acceleration signal and the wear value. Furthermore, the curves for each journal bearing exhibit different values in the initial few cycles due to variations in production. To address this issue, we perform baseline correction by subtracting the minimum median value of each experiment from the other medians of the same experiment, aligning them all to start at the origin after correction.

While using the median of the absolute signal as a metric effectively creates distinct groups with similar wear values for some experiments, its performance degrades in the presence of larger fluctuations, as observed in Experiments 105 and 106 (Figure A1c). Further investigation of the torque curve revealed that these fluctuations were not solely due to sensory malfunctions, but were also visible in the torque measurements. Therefore, we consider the peaks in Experiments 105 and 106 to contain valuable information for predicting wear state.

Next, we tested the α-trimmed mean in combination with the interquartile range as a new metric. The α-trimmed mean is calculated by removing the largest and smallest α-percentile of the ordered data and computing the mean of the remaining values. This approach reduces sensitivity to outliers compared to the regular mean, provided that α is set sufficiently high to include all outliers. For this study, we set α as 10%. Figure A2a presents the results for the same experiment shown in Figure A1c. It is evident that filtering the acceleration measurements alone is insufficient to segregate the wear states adequately. The same holds true when we computed the $L_{1}$ norm with the origin as the reference state (Figure A2b).

In fact, the behavior captured in Figure A1 and Figure A2 reveals an important characteristic of journal bearings. As reported in [17], journal bearings can self-rescue, as evidenced by the decrease in the norm value of Experiment 105 after an increase around cycle 440. Similar behaviors observed in different experiments highlight that considering only the current acceleration values is insufficient. To incorporate memory into the norms, they must be adapted to keep track of the maximum occurred value and recall the wear event that produced the potentially maximum wear from the start of the experiment until the present. Figure A3 explicitly illustrates this process for the $L_{1}$ norm.

After implementing the maximum distance adaptation, representation capabilities of each distance metric is analyzed by calculating the Pearson correlation coefficient between the last cycle distances and the measured wear values after 900 cycles Figure A4: the figure highlights that using either the $L_{1}$ or α-trimmed-mean norm leads to a consistently high Pearson correlation coefficient. While the α-trimmed-mean norm has a slightly higher mean accuracy (92.4%) than the $L_{1}$ norm (91.1%), nonetheless, it necessitates the determination of the α parameter for the given problem. In the current experimental campaign, an α value of 10% was found to provide a robust solution and was used to build the 2D state maps with the CAEs.
